# First-Principles Molecular Dynamics Calculations of the Equation of State for Tantalum

**DOI:** 10.3390/ijms10104342

**Published:** 2009-11-20

**Authors:** Shigeaki Ono

**Affiliations:** 1 Institute for Research on Earth Evolution, Japan Agency for Marine-Earth Science and Technology, 2-15 Natsushima-cho, Yokosuka-shi, Kanagawa 237-0061, Japan; 2 Earthquake Research Institute, University of Tokyo, 1-1-1 Yayoi, Bunkyo-ku, Tokyo 113-0032, Japan; E-Mail: sono@jamstec.go.jp; Tel.: +81-46-867-9631; Fax: +81-46-867-9625

**Keywords:** equation of state, tantalum, high pressure, *ab initio* calculations

## Abstract

The equation of state of tantalum (Ta) has been investigated to 100 GPa and 3,000 K using the first-principles molecular dynamics method. A large volume dependence of the thermal pressure of Ta was revealed from the analysis of our data. A significant temperature dependence of the calculated effective Grüneisen parameters was confirmed at high pressures. This indicates that the conventional approach to analyze thermal properties using the Mie-Grüneisen approximation is likely to have a significant uncertainty in determining the equation of state for Ta, and that an intrinsic anharmonicity should be considered to analyze the equation of state.

## 1. Introduction

Equations of state (EOS) for some elemental metals have been used as an internal pressure gauge in X-ray diffraction high-pressure studies using diamond anvil cell [*e.g.*, 1–3] or multi-anvil press experiments [*e.g.*, 4–6]. Ta is one of good materials for the internal pressure gauge, because the body-centred structure of Ta remains stable up to at least 174 GPa [7], and the melting temperature is higher than most of other metals [8]. The equation of state of Ta has been investigated by previous experimental studies [*e.g.*, 7,9–11]. However, reliable data at high temperature are still not available, because the uncertainty for temperature is non-negligible in the high-pressure experiments. Recently, theoretical studies using first-principles calculations have investigated the physical properties of materials at high pressure and high temperature. We noticed that the scatter of the experimental bulk modulus values at room temperature was much smaller than that obtained from first-principles calculations [7,9,12–17]. This indicates that experiments are more accurate than first-principles calculations for determining the bulk modulus at room temperatures. In contrast, the first-principles molecular dynamics calculations have significant advantages to investigate the physical properties of materials at high temperatures.

In this study, we used density functional theory to investigate the thermal pressure of Ta. We also used the experimental data to determine the room temperature EOS of Ta. The combination of the first-principles molecular dynamics calculations and the high-pressure experiments led us to determine a reliable EOS over a wide range of pressures and temperatures.

## 2. Methods

The first-principles calculations carried out in this study were based on density functional theory using the VASP package [18]. We used the generalized gradient approximation (GGA) [19] for the exchange-correlation functional. The electronic wave functions were expanded in a plane-wave basis set with a cut-off energy of 600 eV, and the electron-ion interactions were described using the projector augmented wave (PAW) method [20,21]. The 5s^2^ and 5p^6^6s^2^5d^3^ of the Ta atom are treated as frozen core and valence electron, respectively. We used a 128-atom supercell with Γ-point Brillouin zone sampling and a time step of 1 fs for the first-principles molecular dynamics simulation at constant volume. Simulations were run in the constant *NVT* ensemble with the Nosé thermostat [22] 5–10 ps after equilibration. Details of our methodology have been given elsewhere [23]. The computation time required to reach equilibration varied between configurations, and depended on the starting atomic positions, velocity, temperature, and pressure. The first-principles molecular dynamics calculations were performed under 50 pressure-volume conditions in this study. The pressure and temperature ranges were 0–100 GPa and 300–3,000 K, respectively. The thermal pressure was calculated at each volume. The total pressure at high temperature and high pressure condition was estimated from room-temperature EOS from experimental data and the thermal pressure from the first-principles molecular dynamics calculations.

## 3. Results and Discussion

### 3.1. Thermal Equation of State

The EOS of a solid can be described in a general form as a functional relationship between the pressure, volume, and temperature as

(1) $$P_{\text{total}}(V,T)=P_{st}(V,300)+P_{th}(V,T)$$

where *P_total_(V,T)* represents the total pressure at volume *V* and temperature *T*. The terms *P_st_(V,300)* and *P_th_(V,T)* represent the static pressure at volume *V* and 300 K and the thermal pressure at volume *V* and temperature *T*, respectively. We determined the thermoelastic parameters for Ta at ambient temperature (300 K) using the Vinet EOS [24], as very high compression data (*V/V_0_* = ∼0.7) was estimated in this study. It is known that the Vinet EOS is suitable for most solids under very high compression. The Vinet EOS is given by the following expression:

(2) Pst=3BT0(VV0)−23[1−(VV0)13]exp{32(BT0'−1)[1−(VV0)13]}

where *B_T0_* is the isothermal bulk modulus at 300 K, *V_0_* is the zero-pressure volume, *V* is the high-pressure volume, and *B_T0_′* is the pressure derivative of *B_T0_*. The thermal pressure EOS [25] was used to evaluate the thermal pressure, *P_th_*. The thermal pressure of the thermal pressure EOS can be written as follows:

(3) $$P_{th}=\alpha B_{T}(T-T_{T0})+{\left(\frac{\partial B_{T}}{\partial T}\right)}_{V}(T-T_{T0})\text{ln}\left(\frac{V_{0}}{V}\right)+{\left(\frac{\partial^{2}P}{\partial T^{2}}\right)}_{V}{\left(T-T_{T0}\right)}^{2}$$

The value of *T_0_* is *T_0_* = 300 K. The parameters of the thermal pressure EOS are α*B_T_*, (*∂B_T_* / *∂T*) *_V_*, and (*∂*^2^ *P* / *∂T*^2^)*_V_*. The thermal expansivity at ambient conditions can be calculated from α*_0_* = (α*B_T_)/B_T0_*.

### 3.2. Equation of State at Ambient Temperature

It is likely that the experimental uncertainty is related to the differential stress in the sample and the credibility of the pressure standard. In the case of diamond anvil cell experiments, the differential stress is accumulated as the pressure increases because the diamond anvils apply a uniaxial compression in the sample chamber. Therefore, the soft materials should be used as the pressure transmitting medium to reduce the differential stress of the sample. It is known that one of best pressure transmitting mediums is helium. Therefore, an experimental data set from Dewaele *et al*. [12] was used to determine the room temperature compression of Ta in this study, because they used the helium pressure transmitting medium. It is known that the influence of the pressure scale on the pressure-volume data is non-negligible. The ruby pressure scale has been frequently used in room temperature compression experiments. Recently, it is known that old ruby scales have a significant uncertainty and typical error is 5%–10% at pressures above 100 GPa [12,14,26,27]. Therefore, the pressure-volume data measured by the old ruby scale in previous experiments should be corrected by the recent reliable ruby pressure scale. Experimental pressures in Dewaele *et al*.’s data set have been corrected by the revised ruby scale of Dorogokupets and Oganov [28].

The bulk modulus, *B_T0_*, of Ta at ambient pressure has been precisely determined by ultrasonic measurements. We used 194 GPa from previous study [9] as the bulk modulus at ambient pressure. Although the bulk modulus obtained by the ultrasonic measurements is likely to be reliable, the pressure derivative of the bulk modulus, *B_T0_′*, from the ultrasonic measurements has a significant uncertainty, because these values were determined at the relatively low pressures. In order to determine the pressure derivative of the bulk modulus at room temperature, we used high-pressure data from the static compression experiments. A least squares fit of the experimental data [12] at 300 K yields *V_0_* = 18.016 Å^3^, *B_T0_* = 194 GPa, and *B_T0_′* = 3.740 using the revised ruby scale of Dorogokupets and Oganov [28]. The volume, *V_0_*, at ambient condition was fixed in the least squares fit.

### 3.3. Thermal Pressure at High Pressure Conditions

**Figure 1.**
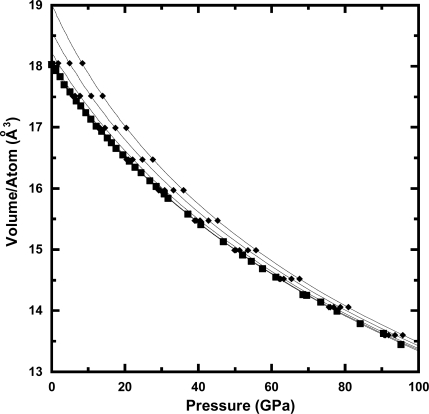
Pressure-volume data for Ta. The squares and diamonds denote the volume from experiments [12] at 300 K and first-principle calculations at temperatures from 300 to 3,000 K. The lines denote the calcula2ted isotherms at temperatures of 300, 500, 1,000, 2,000, and 3,000 K.

**Figure 2.**
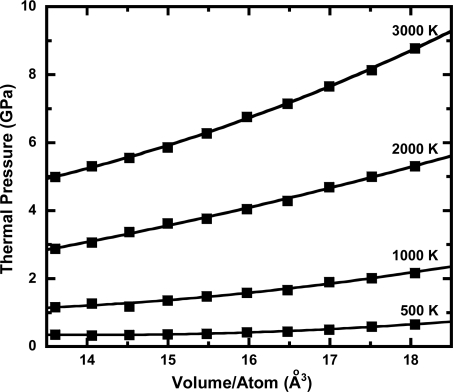
A plot of the thermal pressure (*P_th_*) versus cell volume for Ta. The solid squares denote the calculated thermal pressures from 500 K to 3,000 K.

The first-principle molecular dynamics method has been used to calculate the thermoelastic properties of Ta under extreme high-pressure and high-temperature conditions. Figure 1 shows the *P-V-T* data from both the experiments and calculations used in our study. The results of the fit of our *P*-*V*-*T* data to the thermal pressure EOS are summarized in Table 1. The values of *α_0_*_,_ (*∂B_T_* / *∂T*) *_V_*, and (*∂*^2^ *P* / *∂T*^2^)*_V_* were 1.47 × 10^−5^ (K^−1^), −0.0050 (GPaK^−1^), and 2.11 × 10^−7^ (GPa^2^K^−2^), respectively. Table 2 lists the pressure at selected compressions and temperatures based on the equation of state obtained in this study. Figure 2 shows the thermal pressure, *P_th_*, of Ta versus cell volume. It can be seen that the thermal pressure gradual decreases up to ∼100 GPa as the cell volume decreases. Recently, Liu *et al*. [29] reported that the thermal pressure rapidly increases at pressures higher than 100 GPa using the quasiharmonic approximation. In contrast, previous study based on experimental data [28] estimated that the thermal pressure does not show a big change at wide range of pressure. The inconsistency of thermal pressure at very high compression is still unsolved issue.

**Figure 3.**
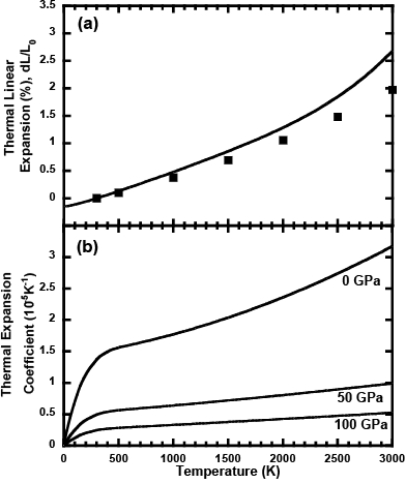
(a) Thermal linear expansion at ambient pressure. The solid line denotes the regression fit from experimental data [30]. The solid squares denote the calculated values from the EOS for Ta in this study. L_0_ is the length at 300 K. (b) Calculated temperature dependence of the thermal expansion coefficient. The results are shown for 0, 50, and 100 GPa.

Figures 3(a) and 3(b) show the thermal expansion properties at ambient pressure and high pressures. The thermal linear expansion at low temperatures calculated from our EOS of Ta is in good agreement with the recommended values from many experimental data [30] in Figure 3(a). However, a small difference between our calculations and experimental data was confirmed at temperatures above 2,000 K, although the uncertainty of experimental data is approximately ±10% above 2,100 K. Therefore, our calculations underestimate the thermal expansion properties compared with experimental data at high temperatures. It is likely that this underestimation is due to the uncertainty of the generalized gradient approximation for the exchange-correlation functional used in this study. The thermal expansion coefficients at different pressures were calculated in Figure 3(b). As the pressure increases, the pressure dependence on the thermal expansion decreases. This is in good agreement with a typical property of condensed materials.

**Figure 4.**
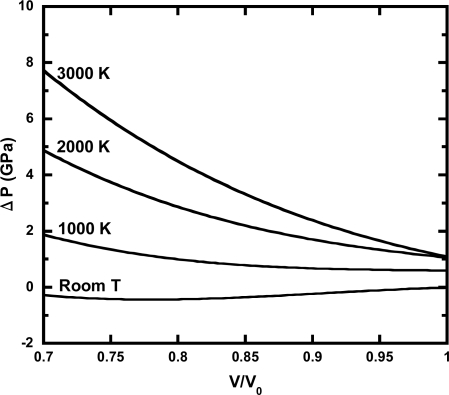
The pressure difference between previous EOS based on experimental data and our EOS combined thermal pressure calculated by first-principles and the room-temperature compression from experimental data. The lines denote pressures of previous EOS [28] minus our EOS on room-temperature, 1,000, 2,000, and 3,000 K isotherms of Ta.

Figure 4 shows the deviations of the calculated pressure on isotherms from previous EOS based on experimental data [28]. The deviation of the room-temperature isotherm was very small, because our EOS used reliable experimental data at room-temperature. In contrast, the deviations of high-temperatures increased as pressure increased. Such deviations have been confirmed in other EOSs of solids (*i.e.*, Au, Pt, MgO) used as pressure standard [28]. It is known that the experimental uncertainties increase rapidly as pressure and temperature increase. As the inconsistency among different experimental data is still an unresolved issue, the uncertainty of EOS determined by experimental data is likely to be significant at high pressures and high temperatures.

### 3.4. Anharmonic Effects

The Mie-Grüneisen-Debye EOS has been frequently used in previous studies on the EOS of solids. The Mie-Grüneisen approximation is valid if the quasiharmonic term is dominant in the thermal pressure. However, it is known that the anharmonic term, which is not included in the Mie-Grüneisen approximation, is not negligible at high temperatures. Therefore, we assessed the effect of the anharmonicity on the EOS for Ta. We used the Grüneisen parameter to investigate the anharmonicity. The effective Grüneisen parameter can be written as follows:

(4) $$\gamma_{\text{eff}}(V,T)=\gamma_{qh}(V)-a(V,T)$$

where *γ_qh_(V)* and *a(V,T)* are the quasiharmonic Grüneisen parameter and the intrinsic anharmonicity term, respectively.

**Figure 5.**
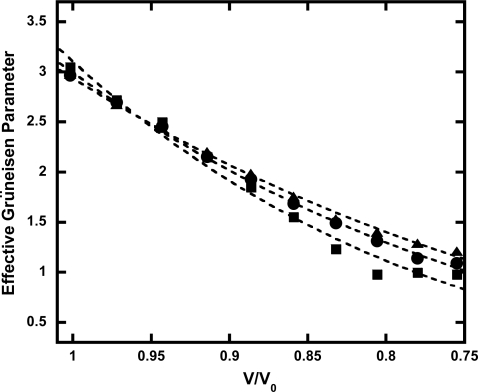
Anharmonic effects on the Grüneisen parameter. The solid squares, circles, and triangles denote the calculated Grüneisen parameter at 1,000, 2,000, and 3,000 K using first-principles molecular dynamics calculations. Errors of calculated values are ∼20%. The dashed lines denote the curve fit using the least square method.

If the anharmonicity is negligible, then the effective Grüneisen parameter does not change at high temperatures. Therefore, we calculated the effective Grüneisen parameter at different volumes and temperatures. The Grüneisen parameter was obtained directly in our calculations from:

(5) $$\gamma =\frac{VP_{th}}{E_{th}}$$

where *E_th_* is the difference of the internal energy. Figure 5 shows the temperature dependence of the effective Grüneisen parameter due to the intrinsic anharmonic effects. The calculated effective Grüneisen parameter at ambient pressure was larger than that reported in previous calculations [29]. At low pressures, a difference in the effective Grüneisen parameter at different temperatures was negligible. However, a significant difference was confirmed at pressures above 30 GPa. Our calculations indicate that the quasiharmonic approximation has a significant uncertainty in the determination of the EOS of Ta at high pressures. For example, the anharmonic thermal pressure contribution was ∼10% at 100 GPa and 3,000 K from the first-principle molecular dynamics calculations. Most of the previous experimental studies on the EOS of solids have not considered the influence of anharmonicity, and the Grüneisen parameter has been assumed to be a function of volume. As the error in the experimental data was considerable, detailed analyses could not be performed in previous studies. In our study, the combination of high-pressure experiments with first-principles molecular dynamics calculations has led to the uncovering of a temperature dependence of the Grüneisen parameter due to the anharmonicity of Ta at high temperatures.

## 4. Conclusions

We have investigated the EOS of Ta, which has a body-centred cubic structure, using the first-principles molecular dynamics method. We used the high-pressure experimental data to determine the compressibility at room temperature, and used the generalized gradient approximation (GGA) and the projector augmented-wave method (PAW) in simulations to calculate the thermal pressure. A Vinet EOS fitted to the room temperature data yielded an isothermal bulk modulus of *B_T0_* = 194 GPa and a pressure derivative of *B_T0_*′ = 3.745. The high-temperature data from the first-principles calculations were fitted to the thermal pressure EOS. The resulting parameters of the thermal pressure, *α_0_*, (*∂B_T_* / *∂T*) *_V_*, and (*∂*^2^*P / ∂T*^2^)*_V_* were 1.47 × 10*^−^*^5^ (K^−1^), −0.0050 (GPaK^−1^), and 2.11 × 10^−7^ (GPa^2^K^−2^), respectively. The temperature dependence of the calculated effective Grüneisen parameters at high pressures indicates that the conventional Mie-Grüneisen approximation is not suitable for the analysis of thermoelastic properties of Ta, and that the intrinsic anharmonicity is non-negligible at high pressures.

## Figures and Tables

**Table 1.** The model thermoelastic parameters of Ta. The Vinet equation of state was used to calculate the parameters of Ta. Key: *B_T0_* = isothermal bulk modulus at 0 GPa and 300 K from ultrasonic experiments [9], *B_T0_′* = first pressure derivative of the bulk modulus, *V_0_* = volume at 0 GPa and 300 K from experiments, *α_0_* = thermal expansion coefficient at 0 GPa.

**Parameter**	
*V_0_* (Å^3^)	18.016
*B_T0_* (GPa)	194
*B_T0_′*	3.740(11)
*α_0_* (10^−5^ K^−1^)	1.47(2)
*(δB_T_/δT)_V_* (GPaK^−1^)	−0.0050(1)
*(δ^2^P/δT^2)^V* (10^−7^GPa^2^K^−2^)	2.11(31)

**Table 2.** Pressure-Temperature-Volume table for Ta from this study. The unit of pressure is GPa.

**1-*V/V_0_***	**300 K**	**500 K**	**1000 K**	**1500 K**	**2000 K**	**2500 K**	**3000 K**
0.00	0.00	0.58	2.10	3.73	5.46	7.30	9.24
0.02	4.07	4.63	6.10	7.67	9.36	11.14	13.04
0.04	8.54	9.08	10.50	12.03	13.66	15.39	17.23
0.06	13.46	13.98	15.35	16.82	18.40	20.08	21.87
0.08	18.88	19.37	20.68	22.10	23.63	25.25	26.99
0.10	24.84	25.30	26.56	27.92	29.39	30.97	32.65
0.12	31.38	31.83	33.04	34.34	35.75	37.27	38.90
0.14	38.60	39.03	40.17	41.42	42.78	44.24	45.80
0.16	46.55	46.96	48.04	49.23	50.53	51.93	53.44
0.18	55.32	55.70	56.72	57.86	59.09	60.43	61.88
0.20	65.00	65.35	66.32	67.38	68.56	69.84	71.22
0.22	75.69	76.02	76.92	77.92	79.03	80.25	81.57
0.24	87.50	87.81	88.64	89.58	90.63	91.78	93.04
0.26	100.59	100.86	101.63	102.51	103.49	104.57	105.76

## References

[1] Fei Y Mao HK Static compresson of Mg(OH)_2_ to 78 GPa at high temperature and constraints on the equation of state of fluid H_2_O J .Geophys. Res 1993 98 11875 11884

[2] Andrault D Fiquet G Guyot F Hanfland M Pressure-induced Landau-type transition in stishovite Science 1998 282 720 772 9784125 10.1126/science.282.5389.720

[3] Ono S Hirose K Kikegawa T Saito Y The compressibility of a natural composition calcium ferrite-type aluminous phase to 70 GPa Phys. Earth Planet. Inter 2002 131 311 318

[4] Irifune T Nishiyama N Kuroda K Inoue T Isshiki M Utsumi W Funakoshi K Urakawa S Uchida T Katsura T Ohtaka O The postspinel phase boundary in Mg_2_SiO_4_ determined by *in situ* X-ray diffraction Science 1998 279 1698 1700 9497283 10.1126/science.279.5357.1698

[5] Ono S Ito E Katsura T Yoneda A Walter MJ Urakawa S Utsumi W Funakoshi K Thermoelastic properties of the high-pressure phase of SnO_2_ determined by *in situ* X-ray observations up to 30 GPa and 1400 K Phys. Chem. Minerals 2000 27 618 622

[6] Wang Y Uchida T Zhang J Rivers ML Sutton SR Thermal equation of state of akimotoite MgSiO_3_ and effects of the akimotoite–garnet transformation on seismic structure near the 660 km discontinuity Phys Earth Planet Inter 2004 143–144 57 80

[7] Cynn H Yoo CS Equation of state of tantalum to 174 GPa Phys. Rev. B 1999 59 8526 8529

[8] Ross M Errandonea D Boehler R Melting of transition metals at high pressure and the influence of liquid frustration: The early metals Ta and Mo Phys. Rev. B 2007 76 184118

[9] Katahara KW Manghnani MH Fisher ES Pressure derivatives of the elastic moduli of niobium and tantalum J. Appl. Phys 1976 47 434 439

[10] Ming L Manghnani MH Isothermal compression of bcc transition metals to 100 kbar J. Appl. Phys 1978 49 208 212

[11] Mitchell AC Nellis WJ Shock compression of aluminum, copper, and tantalum J. Appl. Phys 1981 52 3363 3374

[12] Dewaele A Loubeyre P Mezouar M Equations of state of six metals above 94 GPa Phys. Rev. B 2004 70 094112

[13] Bercegeay C Bernard S First-principles equations of states and elastic properties of seven metals Phys. Rev. B 2005 72 214101

[14] Chijioke AD Nelis WJ Silvera IF High-pressure equations of states of Al, Cu, Ta, and W J. Appl. Phys 2005 98 073526

[15] Orlikowski D Söderlind P Moriarty JA First-principles thermoelasticity of transition metals at high pressure: Tantalum prototype in the quasiharmonic limit Phys Rev B 2006 74 054109.1 054109.10

[16] Foata-Prestavoine M Robert G Nadal M Bernard S First-principles study of the relations between the elastic constants, phonon dispersion curves, and melting temperatures ob bcc Ta at pressures up to 1000 GPa Phys. Rev. B 2007 76 104104

[17] Taioli S Cazorla C Gillan MJ Alfè D Melting curve of tantalum from principles Phys. Rev. B 2007 75 214103

[18] Kresse G Furthmüller J Efficient iterative schemes for ab initio total-energy calculations using a plane-wave basis set Phys. Rev. B 1996 54 11169 11186 10.1103/physrevb.54.11169 9984901

[19] Perdew JP Burke K Ernzerhof M Generalized gradient approximation made simple Phys. Rev. Lett 1996 77 3865 3868 10062328 10.1103/PhysRevLett.77.3865

[20] Blöchl PE Projector augmented-wave method Phys. Rev. B 1994 50 17953 17979 10.1103/physrevb.50.17953 9976227

[21] Kresse G Joubert D From ultrasoft pseudopotentials to the projector augmented-wave method Phys. Rev. B 1999 59 1758 1775

[22] Nosé S A molecular dynamics method for simulations in the canonical ensemble Molec. Phys 1984 52 255 268

[23] Ono S Brodholt JP Alfè D Alfredsson M Price GD *Ab initio* molecular dynamics simulations for thermal equation of state of B2-type NaCl J. Appl. Phys 2008 103 023510

[24] Vinet P Ferrante J Rose J Smith J Compressibility of solids J. Geophys. Res 1987 92 9319 9325

[25] Jackson I Rigden SM Analysis of *P-V-T* data: Constraints on the thermoelastic properties of high-pressure minerals Phys. Earth Planet. Inter 1996 96 85 112

[26] Holzapfel WB Refinement of the ruby luminescence pressure scale J. Appl. Phys 2003 93 1813 1818

[27] Kunc K Loa I Syassen K Diamond under pressure: *ab initio* calculations of the equation of state and optical phonon frequency revisited High Press. Res 2004 24 101 110

[28] Dorogokupets PI Oganov AR Ruby, metals, and MgO as alternative pressure scales: A semienprical description of shock-wave, ultrasonic, X-ray, and thermochemical data at high temperatures and pressures Phys. Rev. B 2007 75 024115

[29] Liu ZL Cai LC Chen XR Wu Q Jing FQ *Ab initio* refinement of the thermal equation of state for bcc tantalum: The effect of bonding on anharmonicity J Phys Condens Matter 2009 21 095408.1 095408.10 10.1088/0953-8984/21/9/095408 21817394

[30] Touloukian TS Kirby RK Taylar RE Desai PD Thermophysical Properties of Matter IFI/Plenum New York, NY, USA 1977 317

